# Urinary Metabolomic Profile of Youth at Risk of Chronic Kidney Disease in Nicaragua

**DOI:** 10.34067/KID.0000000000000129

**Published:** 2023-04-17

**Authors:** Samantha M. Hall, Nathan H. Raines, Oriana Ramirez-Rubio, Juan José Amador, Damaris López-Pilarte, Cristina O'Callaghan-Gordo, Rubén Gil-Redondo, Nieves Embade, Oscar Millet, Xiaojing Peng, Selene Vences, Sinead A. Keogh, Iris S. Delgado, David J. Friedman, Daniel R. Brooks, Jessica H. Leibler

**Affiliations:** 1 Department of Environmental Health, Boston University School of Public Health, Boston, Massachusetts; 2 Division of Nephrology, Department of Medicine, Beth Israel Deaconess Medical Center and Harvard Medical School, Boston, Massachusetts; 3 Barcelona Institute for Global Health, ISGlobal, Barcelona, Spain; 4 Department of Epidemiology, Boston University School of Public Health, Boston, Massachusetts; 5 Faculty of Health Sciences, Universitat Oberta de Catalunya, Barcelona, Spain; 6 Universitat Pompeu Fabra (UPF), Barcelona, Spain; 7 CIBER Epidemiología y Salud Pública (CIBERESP), Madrid, Spain; 8 Precision Medicine and Metabolism Laboratory, CIC bioGUNE, Basque Research and Technology Alliance (BRTA), Bizkaia, Spain; 9 CIBERehd, Instituto de Salud Carlos III, Madrid, Spain; 10 Department of Biostatistics, Boston University School of Public Health, Boston, Massachusetts

**Keywords:** chronic kidney disease, occupational health, Central America, CKDu, CKDnt, Mesoamerican nephropathy, pediatric kidney disease, adolescent, epidemiology, metabolomics, Nicaragua, occupational health

## Abstract

**Key Points:**

- Urinary concentrations of glycine, a molecule associated with thermoregulation, were elevated among youth from a high-risk region for chronic kidney disease of non-traditional etiology (CKDnt).
- Urinary concentrations of pyruvate, citric acid, and inosine were lower among youth at higher risk of CKDnt, suggesting renal stress.
- Metabolomic analyses may shed light on early disease processes or profiles or risk in the context of CKDnt.

**Background:**

CKD of a nontraditional etiology (CKDnt) is responsible for high mortality in Central America, although its causes remain unclear. Evidence of kidney dysfunction has been observed among youth, suggesting that early kidney damage contributing to CKDnt may initiate in childhood.

**Methods:**

Urine specimens of young Nicaraguan participants 12–23 years without CKDnt (*n*=136) were analyzed by proton nuclear magnetic resonance spectroscopy for 50 metabolites associated with kidney dysfunction. Urinary metabolite levels were compared by, regional CKDnt prevalence, sex, age, and family history of CKDnt using supervised statistical methods and pathway analysis in MetaboAnalyst. Magnitude of associations and changes over time were assessed through multivariable linear regression.

**Results:**

In adjusted analyses, glycine concentrations were higher among youth from high-risk regions (*β*=0.82, [95% confidence interval, 0.16 to 1.85]; *P* = 0.01). Pyruvate concentrations were lower among youth with low eGFR (*β*= −0.36 [95% confidence interval, −0.57 to −0.04]; *P* = 0.03), and concentrations of other citric acid cycle metabolites differed by key risk factors. Over four years, participants with low eGFR experienced greater declines in 1-methylnicotinamide and 2-oxoglutarate and greater increases in citrate and guanidinoacetate concentrations.

**Conclusion:**

Urinary concentration of glycine, a molecule associated with thermoregulation and kidney function preservation, was higher among youth in high-risk CKDnt regions, suggestive of greater heat exposure or renal stress. Lower pyruvate concentrations were associated with low eGFR, and citric acid cycle metabolites, such as pyruvate, likely relate to mitochondrial respiration rates in the kidneys. Participants with low eGFR experienced longitudinal declines in concentrations of 1-methylnicotinamide, an anti-inflammatory metabolite associated with anti-fibrosis in tubule cells. These findings merit further consideration in research on the origins of CKDnt.

## Graphical abstract

**Figure:**
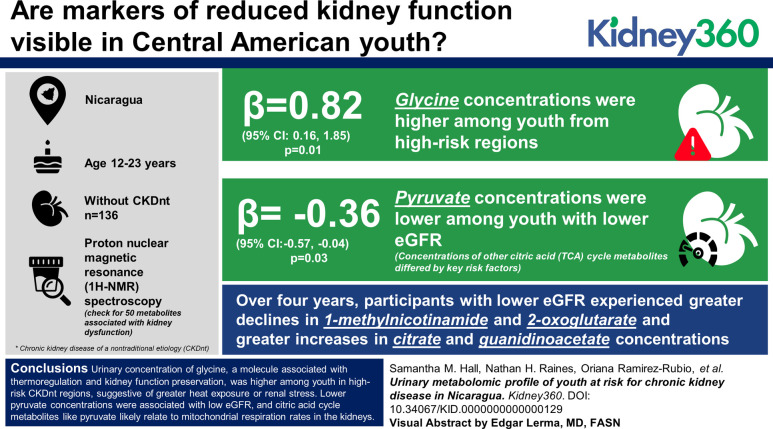

## Introduction

An ongoing epidemic of CKD of nontraditional etiology (CKDnt) poses a significant public health crisis in Central America. The disease, also known regionally as Mesoamerican nephropathy, is a leading cause of death in the area and primarily affects young men between 20 and 50 years working in agriculture and other outdoor manual labor occupations along the Pacific coast of Central America.^1^ Despite more than a decade of research, the etiology of this deadly disease remains elusive, although most researchers agree that the cause is likely multifactorial.^2^

Heat stress as a core risk factor of CKDnt has emerged as a leading hypothesis, although the pathogenesis of extreme heat as an initiator or progression factor of CKDnt remains under investigation.^3,4^ Exposures to agrichemicals, heavy metals, and infectious agents have been proposed and studied as risk factors, although to date these associations are unclear.^4,5^ While CKD in high-income countries typically occurs in older individuals with chronic conditions, such as hypertension and diabetes, individuals with CKDnt usually present much younger, not uncommonly as young as 20 years.^1,2^ This observation suggests that early-life exposures may play a role in disease development or progression, perhaps increasing susceptibility to kidney disease in the context of later-life exposures.^6,7^

To date, there are limited data on pediatric kidney disease in the region, although our work and that of others indicate an elevated prevalence of kidney injury biomarkers among youth without occupational experience, suggesting that prenatal, community, and/or genetic exposures may contribute to disease.^6–8^ Specifically, we observed elevated levels of urinary kidney injury biomarkers neutrophil gelatinase-associated lipocalin (NGAL), *N*-acetyl-_D_-glucosaminidase, and IL-18, in our studies of Nicaraguan youth compared with referent populations, suggesting possible kidney injury in this population.^6,8^

Despite these observations, assessments to date have not been designed to identify pathophysiologic processes or specific exposures associated with pediatric disease in this population. Metabolomic analyses allow for broad investigations into function, disease pathology, and exposure profiles.^9,10^ Urinary metabolites reflect gene expression, physiological and biological processes, diet, chemical exposures, and microbial activity.^10,11^ Metabolomic analysis has high applicability to organ-specific pathologies; urinary metabolites have been used to elucidate CKD stages and predict onset of kidney dysfunction and disease.^12,13^ As such, metabolomic analysis may be useful in evaluating early signals of kidney damage among youth and improving our understanding of the etiology of CKDnt.

The primary objective of this study was to assess urinary metabolic features associated with reduced kidney function in children and adolescents. Secondary objectives were to evaluate whether other key risk factors of CKDnt were associated with distinct metabolic profiles or with individual-level changes in urinary metabolite concentrations between study years. This untargeted metabolomic analysis aimed to identify patterns among risk groups and generate hypotheses for future exploration.

## Methods

### Study Design

Nicaraguan youth were recruited at their schools through on-site visits and parent information sessions. Four schools were selected as recruitment sites: two from an area with high adult prevalence of CKDnt (Chichigalpa in the department of Chinandega, with a 2017 CKD prevalence of 20.5% [95% confidence interval (CI), 16.7 to 24.6]), one from an area of low-to-moderate CKD prevalence (Masaya, 15.5% CKD [11.7, 19.9] in 2017), and one from a high-altitude region with low CKDnt prevalence (Jinotega, 6.8% CKD [4.5, 9.8] in 2017).^14^ Masaya and Jinotega schools served as the referent populations with lowered *a priori* CKDnt risk. Inclusion criteria are described elsewhere.^8^ Parental consent and participant assent were obtained for all participants. All study protocols were approved by the Boston University Medical Campus Institutional Review Board and by the Ethical Committee of the Nicaraguan Ministry of Health.

Study participants provided a clean-catch urine specimen at baseline in 2011 and again in 2015, when a blood sample was also collected. Specimens were subsequently stored frozen at −80°C until analysis for this study. In-person interviews (30 minutes) were conducted both years. The CKD in Children Under 25 (CKiD U25) serum creatinine–based equation was used to estimate the serum glomerular filtration rate (eGFR) given the ages of study participants (Supplemental Methods).^15^ Urine IL-18 and NGAL were analyzed as described previously in 2011 samples.^8^ We evaluated whether urine concentrations of metabolite levels from participants with a low eGFR consistent with early kidney dysfunction in this age group (≤90 ml/min per 1.73 m^2^) differed from those with normal eGFR (>90 ml/min per 1.73 m^2^).^16^ In secondary analyses, we compared metabolite levels among other prespecified groups of interest: residence in high-risk regions for CKDnt (Y/N), history of CKDnt in the immediate family (parents or siblings; Y/N), older and younger than 18 years, male versus female, and quartile of urinary NGAL and IL-18 concentrations (highest quartile versus lower three quartiles). We analyzed data from two collection points both cross-sectionally at each year (2011 and 2015) and longitudinally, considering the individual-level change in metabolite concentrations between these years as the end point.

### Metabolite Quantification by ^1^H-NMR

In this untargeted analysis, urine specimens from all participants were analyzed at 300K using a 600 MHz in vitro diagnostic regulation (IVDr) spectrometer (Bruker BioSpin, Germany) with a tempered SampleJet automatic sample changer mounted on it and a double resonance broadband probe (BBI) head with a z gradient coil and BOSS-III shim system.^17^ Briefly, samples were stored at −80°C until analysis. Aliquots were centrifuged at 6000 rpm for 5 minutes at 4°C, and then, 630 *μ*l of the supernatant was transferred into a 1.5 ml tube. Subsequently, 70 *μ*l of a phosphate buffer was added in the same microcentrifuge tube to minimize pH variation. The mix of urine and buffer was briefly vortexed, and 600 *μ*l of the mixture was transferred into a 5-mm nuclear magnetic resonance tube. Two complementary experiments were recorded per sample: a standard one-dimensional (1D) ^1^H spectrum with water presaturation and 10s re-equilibration delay for metabolite quantification and a two-dimensional (2D) J-resolved (Jres) to help metabolite identification. Bruker IVDr Quantification in URine B.I.Quant-UR b software (v. 1.0.0) was used to automatically identify and quantify up to 50 urine metabolites in this untargeted analysis.

### Statistical Analyses

#### Individual Metabolite Analyses

Urine metabolites and biomarkers were standardized by urine creatinine and body weight as a conservative estimate.^18–21^ Corrected metabolites were log-transformed and then mean centered to improve normality. Metabolite levels below the nuclear magnetic resonance limit of detection were replaced with one-fifth of the minimum positive value for each metabolite. We reran models excluding metabolites with more than 80% missingness to consider the overall effect of missing data on our conclusions.

In MetaboAnalyst 5.0, differences between prespecified groups of interest were analyzed using supervised partial least squares–discriminant analysis (PLS-DA) and random forest. Machine learning model parameters were automatically optimized per model in MetaboAnalyst. Permutation tests based on separation distance were conducted to assess whether separation observed between designated groups was greater (*P*-value <0.05) than separation by random grouping in PLS-DA. Analyses were conducted both in the overall population and in sex-based strata.

#### Pathway Analysis

In MetaboAnalyst, groups were compared by metabolic pathway differences, with metabolites grouped by pathway on the basis of the Kyoto Encyclopedia of Genes and Genomes. We used a Holm-Bonferroni adjusted *P*-value for the difference in pathway-clustered metabolites between groups using the GlobalTest.^22^ Pathways with both an effect score ≥0.1 and a Holm adjusted *P*-value <0.05 were identified as key features differentiating groups. Within key pathways, metabolites reported at levels different than expected through a GlobalTest (*P* < 0.05) and as important to the metabolic pathways (importance score ≥0.1) were identified as key individual metabolites.

#### Multivariable Regression of Key Metabolites

Key metabolites identified through supervised analyses in MetaboAnalyst were modeled in multivariable linear regression models in R Studio (version 1.4.1717) to control for potential confounders and expected physiological trends related to sex and age groups. Models predicting individual metabolite concentrations were adjusted for the following *a priori* risk factors: sex, age, familial history of CKDnt, regional CKDnt prevalence, and eGFR status. In models with a taurine outcome, we included data on consumption of a rehydration drink, bolis, under a hypothesis that participants consuming these drinks may have increased levels of urinary taurine. In addition, we evaluated the longitudinal end point of individual key metabolite changes from 2011 to 2015 in relation to the predictors as earlier using adjusted linear regressions. Beta coefficients and 95% CIs were back-transformed by exponentiating the coefficient and subtracting one.

## Results

We analyzed urine specimens from 136 participants who provided samples in both 2011 and 2015. This subset reflected a random sample selected to match region, age, and sex of our study population in 2015 (Supplemental Figure S1). Of participants, 64 (46%) were male and 69 (51%) were from a CKDnt high-risk region (Table 1). In 2015, the median age was 19 years, with 108 participants (79%) aged 18 years or older. There were eight participants (5.8%) with eGFR values ≤90 ml/min per 1.73 m^2^ in 2015. Of this group, five (63%) were male. Our untargeted method identified 50 urinary metabolites (Supplemental Table S1), and exploratory analyses excluding metabolites with >80% missingness (8 of 50 metabolites, results not shown) produced the same results as analyses that imputed missing metabolites.

**Table 1:** Characteristics of Nicaraguan adolescent study population (*n*=136)

Characteristic	Study Population, 2015 (*n*=136)
**Sex**	
Male (*n*, %)	63/136 (46.3%)
Body weight, kg (median, IQR)	60.5 (19.3)
**Age**	
Range (in yr)	14–23
Median, IQR (in yr)	19 (2)
≥18 years (*n*, %)	108/136 (79.4%)
High-risk region for adult MeN^a^ (*n*, %)	69/136 (50.7%)
Familial history of MeN^b^	19/135 (14.1%)
eGFR^c^ ≤90 ml/min per 1.73 m^2^	8/136 (5.9%)
Have consumed bolis at work^d^	11/49 (22.4%)

IQR, interquartile range; MeN, Mesoamerican nephropathy.

a Participants originating from the town of Chichigalpa in the department of Chinandega are considered high-risk; participants originating from departments Masaya and Jinotega are considered lower risk.

b Familial history was determined using a questionnaire as to whether the participant has an immediate family member who has been diagnosed with CKD.

c eGFR calculated with CKiD Under 25 equation.

d Only participants who were employed at the time of the interview (*n*=49) were asked whether they consume bolis at work.

### Pairwise Analyses

#### Low versus Normal eGFR

There was no significant difference between individuals with low (≤90 ml/min per 1.73 m^2^) versus normal (>90 ml/min per 1.73 m^2^) eGFR in pairwise PLS-DA or RF analyses, and pathway analysis showed no pathways of a high effect. These trends held when eGFR groups were stratified by sex.

#### Differences in Individual Metabolites by Secondary Characteristics

**Figure 1:**
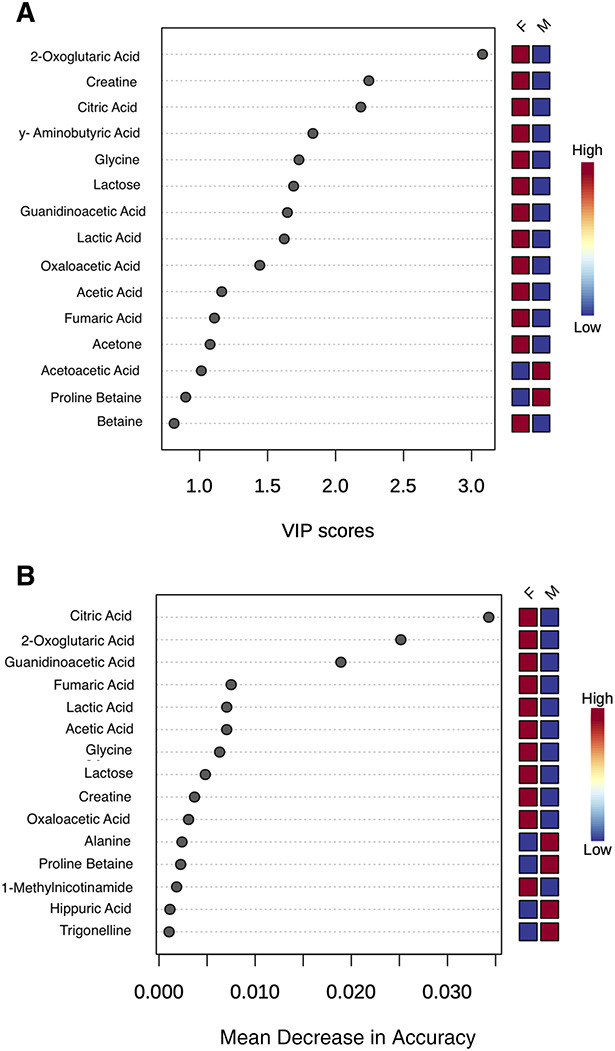
**Supervised analysis of creatinine- and body weight–standardized metabolite patterns from 2015 by sex groups was performed through partial least squares–discriminant analysis (PLS-DA) and random forest (RF).** (A) shows important variables from PLS-DA and (B) shows important variables from RF. Important individual metabolites were identified by calculating VIP scores for the first component of PLS-DA models and mean decrease in accuracy values in RF. PLS-DA, partial least squares–discriminant analysis; RF, random forest; VIP, variable importance in projection.

We observed significant differences in urinary metabolites by sex in PLS-DA and RF analyses (PLS-DA permutation *P* < 0.01; Supplemental Figure S2). A plurality of important metabolites driving sex group separation in PLS-DA are involved in the tricarboxylic acid cycle (also known as the citric acid or TCA cycle) (Figure 1). RF predicted 2015 male versus female metabolomes with 25% and 19.2% errors, respectively, with a 0.213 out-of-bag error and a 0.57 Matthew correlation coefficient (Supplemental Figure S2). This was the most successful modeling of separation between all risk variables. There was no significant separation by PLS-DA or RF analysis by age, family risk, risk region of origin, or NGAL or IL-18 levels (*P*-values>0.05).

#### Pathway Analysis

Participants grouped by age, family history, and NGAL quartile did not show significant differences in metabolite patterns in pathway analysis when considered both overall and stratified by sex. In analyses of male participants from regions of high versus low CKDnt prevalence, glutathione metabolism met criteria for statistical significance (Holm adjusted *P* = 0.03), but was marginal in terms of impact (impact score=0.09, Table 2). Within the glutathione pathway, male participants from high-risk regions had lower urinary glycine levels compared with male participants in low-risk regions in 2015 (importance=0.25, *P* < 0.01, Table 3). Taurine and hypotaurine metabolism was identified as high impact in comparisons between participants in the highest quartile of IL-18 versus those with lower IL-18 in 2011 (impact score=0.43, Holm adjusted *P* < 0.01, Table 2). Urinary taurine was lower among participants with IL-18 levels in the highest quartile compared with those in the bottom three quartiles (importance score=0.43, *P* < 0.001, Table 3), although this trend did not hold when IL-18 was evaluated as quartiles and stratified by sex.

**Table 2:** Pathways of significance and suggested significance across risk factors, assessed for 2011 and 2015 specimens

Risk Variable	Pathway	Total Compounds	Hits	Holm-Bonferroni Adjusted *P*	Impact
Sex	Alanine, aspartate, and glutamate metabolism	28	8	<0.001	0.23
	Citrate cycle (TCA)	20	6	<0.001	0.38
	Glyoxylate and dicarboxylate metabolism	32	6	<0.01	0.17
	Pyruvate metabolism	22	5	<0.01	0.27
	Glycolysis/Gluconeogenesis	26	5	0.01	0.13
	Butanoate metabolism	15	4	0.01	0.14
	Arginine biosynthesis	14	3	0.01	0.08
	Glycine, serine, and threonine metabolism	33	7	0.01	0.49
	Arginine and proline metabolism	38	5	0.02	0.12
Risk region—male participants only	Glutathione metabolism	28	1	0.03	0.09
IL-18	Taurine and hypotaurine metabolism	8	1	0.003	0.43

**Table 3:** Key metabolites identified from univariate pathway analysis using 2015 and 2011 metabolites

Metabolite	KEGG C ID	Importance Score	*P*-Value^a^	Pathway	Risk Variable of Significant (or Suggested) Importance
Acetate	C00033	0.1	<0.01	Pyruvate metabolism	Sex
Citrate	C00158	0.1	<0.001	Citrate cycle	Sex
Fumarate	C0012	0.03	0.01	Citrate cycle	Sex
Glycine	C00037	0.3	0.02	Glycine, serine, and threonine metabolism; glyoxylate and dicarboxylate metabolism	Sex
		0.3	<0.01	Glutathione metabolism	Risk region (sex stratified)
Guanidinoacetate	C00581	0.03	<0.001	Glycine, serine, and threonine metabolism	Sex
1-Methylnicotinamide	C02918	0.1	0.01	Nicotinate and nicotinamide metabolism	Sex
Oxaloacetate	C00036	0.1	0.2	Citrate cycle, alanine; aspartate, and glutamate metabolism	Sex
2-Oxoglutarate	C00026	0.1	<0.001	Citrate cycle; alanine, aspartate, and glutamate metabolism	Sex
Pyruvate	C00022	0.046	0.2	Citrate cycle	Sex
Taurine	C00245	0.4	<0.001	Taurine and hypotaurine metabolism	IL-18

KEGG, Kyoto Encyclopedia of Genes and Genomes.

a *P*-value generated using Holm-Bonferroni adjustment.

In pathway analysis comparing male and female participants, the glycine, serine, and threonine metabolism pathway was most impactful (*P* < 0.001; impact score=0.49). Glycine was the most relevant metabolite (*P* < 0.05). Other high-effect pathways also identified glycine as an important metabolite in 2011 and 2015 samples (Tables 2 and 3).

### Regression Analysis

#### Cross-Sectional Data

In fully adjusted models, glycine was positively associated with high-risk CKDnt region (*ß*=0.82 [95% CI, 0.16 to 1.85], *P* = 0.01). Pyruvate was negatively associated with low eGFR (*ß*=−0.36 [95% CI, −0.57 to −0.04], *P* = 0.03), and oxaloacetate was positively associated with low eGFR (*ß*=1.5 [0.15, 4.44], *P* = 0.02; Table 4). Taurine was positively associated with bolis consumption for the subset reporting current employment (*n*=49; *ß*=1.93 [95% CI, 0.66 to 4.18], *P* < 0.01; Table 4, Supplemental Table S2). Concentrations of acetate, citrate, fumarate, glycine, guanidinoacetate, 1-methylnicotinamide, oxaloacetic acid, and 2-oxoglutarate were lower in male participants compared with female participants in adjusted regressions (Table 4).

**Table 4:** Select results of adjusted linear regression models run for ten key metabolites identified from individual metabolite and pathway analyses of 2015 data, as well as results from longitudinal regression analysis with the dependent variable calculated as the change in metabolite levels from 2011 to 2015

Metabolite	Dependant variable	*β* (95% CI), *P*-Value
**Cross-sectional urinary metabolite concentrations, 2015**		
Acetate	Sex^a^	−0.27 (−0.42 to −0.09), <0.01
Citrate	Sex	−0.47 (−0.61 to −0.28), <0.01
Fumarate	Sex	−0.26 (−0.44 to −0.02), 0.04
Glycine	Sex	−0.37 (−0.59 to 0.03), 0.04
	Risk region^b^	0.82 (0.16 to 1.85), 0.01
Guanidinoacetate	Sex	−0.36 (−0.48 to −0.21), <0.01
1-Methylnicotinamide	Sex	−0.18 (−0.31 to −0.02), 0.03
2-Oxoglutarate	Sex	−0.58 (−0.77 to −0.23), <0.01
Oxaloacetate	Sex	−0.34 (−0.54 to −0.06), 0.02
	eGFR^c^	1.5 (0.15 to 4.44), 0.02
Pyruvate	eGFR	−0.36 (−0.57 to −0.04), 0.03
Taurine	Bolis consumption^d^	1.93 (0.66 to 4.18), <0.01
**Change in urinary metabolite concentrations between 2011 and 2015**		
Citrate	eGFR	20.38 (16.46 to 23.19), <0.001
Guanidinoacetate	eGFR	11.23 (9.49 to 12.55), <0.001
1-Methylnicotinamide	Sex	−0.11 (−0.20 to −0.008), 0.03
	eGFR	−0.39 (−0.64 to −0.14), <0.01
2-Oxoglutarate	eGFR	−1.16 (−1.94 to −0.38), <0.01
Taurine	Bolis consumption	6.21 (3.25 to 7.63), 0.02

All regressions adjusted for age, eGFR≤ or >90 ml/min per 1.73 m^2^, familial risk of CKD, and risk region of origin. Bolis consumption only added to taurine regressions because of physiological relevance. CI, confidence interval.

a Female sex as referent.

b Low-risk region of origin as referent.

c eGFR >90 ml/min per 1.73 m^2^ as referent.

d Never consuming bolis as referent.

#### Longitudinal Data

In adjusted models considering the longitudinal change in metabolite concentrations over time, those with low eGFR had a greater increase in both citric acid and guanidinoacetate compared with those with normal eGFR from 2011 to 2015 (Table 4). Those with low eGFR showed greater declines in 1-methylnicotinamide and oxoglutarate levels compared with those with normal eGFR. Bolis consumers (*n*=49, Supplemental Table S2) showed a greater increase in taurine levels compared with those who never consume bolis over time. We did not find significant associations between longitudinal change in other key metabolite levels.

## Discussion

We evaluated urinary metabolic profiles associated with risk factors of CKDnt among young Nicaraguan participants to assess whether we could identify indicators of early or subclinical renal dysfunction or increased disease risk. Our analyses suggested that urinary metabolites glycine, pyruvate, citric acid, guanidinoacetate, fumarate, oxoglutaric acid, oxaloacetic acid, 1-methylnicotinamide, and acetate may differentiate across some risk characteristics in this population at high risk of CKDnt, including lower eGFR, region, and sex. We did not otherwise observe differences in metabolic profiles.

### Differences by Renal Function

After adjusted analysis, we found changes in TCA cycle metabolites—pyruvate, oxaloacetate, citrate, and oxoglutarate—for those with lowered eGFR. The TCA cycle is central to cellular energy metabolism by providing key inputs for the mitochondrial electron transport chain. It also plays an important role in cellular signaling. Kidney function is closely linked to mitochondria metabolism, and decreased TCA cycle activity and mitochondrial dysfunction have been associated with kidney injury and disease.^13,23,24^

Prior metabolomic studies have identified decreased pyruvate and TCA cycle metabolites in kidney disease.^13,25^ In our prior study, decreased TCA cycle metabolites are a urinary metabolic feature distinguishing adults engaged in agricultural work associated with high CKDnt risk in Central America.^26^ These findings parallel those of the current study. Pyruvate is the end product of glycolysis and a key substrate for the TCA cycle. In a rodent study, rats with ischemia and glycerol-induced AKI observed renal corticoid pyruvate depletion as a potential secondary mediator of AKI events.^25^ Oxaloacetate, a component metabolite of the TCA cycle, was positively associated with low eGFR and negatively associated with male sex in our study.^24^ Glutamic oxaloacetate transaminase, an enzyme that synthesizes oxaloacetate, has been found to decrease with CKD progression.^27^

In our longitudinal analysis, those with low eGFR showed a larger increase in citric acid compared with normal eGFR from 2011 to 2015, although we observed lower citrate in male versus female participants cross-sectionally in 2015. The acid form of citrate, citric acid, is a central molecule of the TCA cycle. Urinary citrate levels are most studied in relation to the development of kidney stones; low citrate has been shown to increase risk of kidney stones, but less is known about its relationship to CKD.^28^ A gene expression and metabolomic analysis found decreased urinary citrate and 2-oxoglutarate for non-diabetic patients with CKD compared with controls, with TCA disruptions driving pathway differences.^13^ We similarly detected decreased 2-oxoglutarate in our low eGFR group in adjusted analysis, along with lower 2-oxoglutarate in male participants compared with female participants. 2-Oxoglutarate is another component of the TCA cycle, with important roles in cellular signaling as well.^29^ An independent nested case-control study found decreased urinary citrate in those with progressive CKD compared with healthy controls.^30^ This study also found increased levels of other urinary TCA cycle metabolites, including fumarate.^30^ In our study, we did not observe an association between fumarate and eGFR, but fumarate levels were lower in male participants than female participants.

Our findings support differences in TCA metabolite patterns on the basis of kidney function and sex, both cross-sectionally and longitudinally. The direction of our observed trends should be interpreted with caution; we are unable to conclusively link the observed decline in TCA metabolites with simultaneous kidney function because of limited statistical power with small numbers of those with eGFR ≤90 and potential expected differences in physiology between the sexes. Mitochondrial respiration rates warrant further analysis in the context of CKDnt.

In our longitudinal analysis, guanidinoacetate and 1-methylnicotinamide were also related to kidney function. Guanidinoacetate, produced in the renal cortex, is the precursor to creatine. It has been most studied in relation to creatinine metabolism and neurological disorders through guanidinoacetate methyltransferase deficiency.^31^ In a follow-up study of children with CKD, decreased eGFR was associated with decreased urinary guanidinoacetate.^32^ While we observed increased guanidinoacetate between 2011 and 2015 for those with lower eGFR, our study also recorded decreased levels for male participants in cross-sectional analysis.

1-Methylnicotinamide is an anti-inflammatory agent produced from the metabolism of nicotinamide by nicotinamide *n*-methyltransferase. It has been explored in therapies to reduce inflammation in the kidneys, liver, and skeletal muscles.^33^ An investigation of oxidative stress and proximal tubule damage found that mice overloaded with free fatty acid–bound albumin had decreased oxidative stress and increased kidney health outcomes when treated with 1-methylnicotinamide.^34^ Another animal study found that exogenous 1-methylnicotinamide reduced fibrosis and inflammation in fibrotic kidneys with tubular injury, likely by negative regulation of the TGF-*β*1/Smad 3 pathway.^33^ Because of its role in nicotinamide metabolism, 1-methylnicotinamide levels may also be relevant to NAD+ metabolism, a key area of investigation in kidney disease.^35^ Our observation of decreased 1-methylnicotinamide longitudinally for those with lower eGFR and male participants may relate to renal inflammation and increased oxidative stress as our population ages.

#### Regional Differences

In adjusted analyses, we observed higher levels of glycine among participants from high-risk versus low-risk regions. This is the opposite trend observed in male-restricted pairwise analysis; this change is likely an artifact of a very small sample size in sex-stratified unadjusted analysis. Glycine, a nonessential amino acid, is involved in the production of macromolecules, such as DNA and phospholipids, although its role in kidney disease remains somewhat inconclusive.^36^ Animal studies suggest a renal protective quality of glycine and the possibility of low glycine as associated with increased risk of kidney dysfunction.^37^ A case-control study of CKD cases with age- and sex-matched controls in the Framingham Offspring cohort showed higher levels of urinary glycine associated with decreased incident risk of kidney disease.^36^ Serum glycine is also a known thermoregulator, with increased levels having a vasodilatory effect enhancing heat dissipation.^38^ Higher glycine levels in the at-risk regions of our study may reflect differences in heat exposure at the time of specimen collection.

Differences in metabolite patterns by region of origin may be expected. Other work by our group similarly showed important regional differences in metabolite patterns in adults at risk of CKDnt when comparing agricultural workers living in Nicaragua and Spain.^26^ A multicenter metabolomic study in China has identified differences by residential region greater than differences observed within each center.^39^ While metabolic differences in relation to geography could represent exposures, cultural or community practices, or genetic factors, further research is needed to understand the relevance of our findings on the basis of region of origin and kidney function predictions.

#### Behavioral Differences

Urinary taurine was lower in individuals with higher levels of IL-18 in unadjusted analyses, although these trends did not hold after statistical adjustment. We show increased taurine for individuals who self-reported bolis consumption both cross-sectionally and longitudinally after adjustment. Differences in taurine metabolism have been shown to be associated with kidney disease progression in both animal and human studies.^40–43^ While taurine may be an endogenous metabolite, it is also a component of various popular energy drinks, including rehydration solutions provided to sugarcane workers in Nicaragua (bolis).^40,44^ Acute consumption of energy drinks is identified in the literature as a risk factor of AKI episodes, sometimes in conjunction with alcohol consumption.^45,46^ While energy drinks may be consumed to protect the kidneys in the presence of dehydration in occupational settings in Nicaragua, attention should be directed as well to high consumption levels of these drinks.

#### Sex Differences

In pairwise comparisons, group differentiation was primarily driven by sex categories. This difference may be a function of normal adolescent development, although this distinction remains important in the context of CKDnt because of the presentation of the disease predominantly in males.^47^ Lower levels of acetate, citrate, fumarate, glycine, guanidinoacetate, 1-methylnicotinamide, 2-oxoglutarate, and oxaloacetate were associated with male sex in fully adjusted models. These metabolites also drove sex differentiation in pairwise comparisons and are discussed above in relation to eGFR status and kidney function through the TCA. Our observation of decreased acetate in male participants, a precursor to the TCA cycle input acetyl coA, is complementary to the trends of potentially impaired mitochondrial metabolism in those with decreased eGFR, although little evidence was available to conclusively link acetate to kidney function predictions.^48^

Further research is needed to evaluate metabolite pattern differences between male and female participants in our population, both as baseline measures of health and in relation to changes in kidney function. This exploratory analysis has generated further molecular evidence of differences between male and female participants, especially as related to mitochondrial metabolism. Future, larger studies may address whether these trends relate to kidney function decline, a crucial question for this high-risk population.

#### Limitations

This is a small study with an exploratory framework, limited by small sample size and a relatively small number of metabolites. In addition to our small sample size of eight individuals with low eGFR, most of them were male (*n*=5/8). Owing to our noted differences in metabolomic patterns by sex groups mostly driven by TCA metabolites, it is possible that our findings of metabolic differences by eGFR status are an artifact of the separation in metabolic patterns by sex. Spot urine measurements, as used here in cross-sectional analysis, are limited in their interpretability of long-term processes and pathophysiology. Our longitudinal assessment attempted to further investigate longer term processes, but was limited by the use of only two time points. Because a significant proportion of children in rural Nicaragua receive only primary school education and because the adult prevalence of CKDnt is correlated with lower educational attainment, our study may have had lower representation of adolescents at the highest risk of development of CKDnt because we recruited participants from secondary schools. Metabolites of relevance may have different half-lives in the body, reflecting a bias toward chronically shed compounds. Urine metabolites may reflect several processes, including cellular metabolism, interchanges between cells and blood or urine, and filtration and subsequent secretion or reabsorption between vascular and urinary spaces; our study design is unable to distinguish the relative contribution of each of these factors. Proton nuclear magnetic resonance spectroscopy is relatively insensitive to small variations in metabolite levels.^49^ Use of the CKD in Children Under 25 equation to estimate eGFR may impose some misclassification, but it remains the most commonly applied clinical method of addressing kidney dysfunction in adolescents and young adults.^15^

#### Conclusions

In our analysis of urinary metabolomic profiles of youth at risk of CKDnt in Nicaragua, we identified differences in acetate, citrate, fumarate, glycine, guanidinoacetate, 1-methtylnicotinamide, oxaloacetate, 2-oxoglutarate, and pyruvate among youth when grouped by various factors believed to increase risk of disease development. Metabolomic analyses may elucidate early disease processes or highlight increased risk of disease development, posing opportunities for screening and informing etiologic research for CKDnt.

## Disclosures

D.R. Brooks reports the following: Advisory or Leadership Role: Consortium on the Epidemic of Nephropathy in Central America and Mexico, Executive Board Member, unpaid. D.J. Friedman reports the following: Consultancy: Vertex; Ownership Interest: Apolo1bio; Research Funding: Vertex; Honoraria: Sanofi; Patents or Royalties: BIDMC; Advisory or Leadership Role: Vertex FSGS advisory board; and Other Interests or Relationships: Coinventor on patents related to APOL1 diagnostics and therapeutics. O. Millet reports the following: Honoraria: I have been paid honoraria by ATLAS Molecular Pharma S.L. currently or during the past 24 months; and Advisory or Leadership Role: CEO at ATLAS Molecular Pharma S.L. N.H. Raines reports the following: Consultancy: Anapol Weiss and Morgan & Morgan; and Advisory or Leadership Role: La Isla Network—unpaid and Consortium for the Epidemic of Nephropathy in Central America and Mexico—unpaid. All remaining authors have nothing to disclose.

## Supplementary Material

## Data Availability

All data are included in the manuscript and/or supporting information: Deidentified data used in analyses are provided as a Supplemental File.

## Supplemental Material

1. Supplemental Methods. Serum creatinine measurement for use in CKiD eGFR calculation.

2. Supplemental Figure S1. Flowchart of cohort participants.

3. Supplemental Table S1. Urine metabolites (*n*=50) measured for each participant through ^1^H-NMR.

4. Supplemental Figure S2. Partial least square–discriminant analysis (PLS-DA) and random forest (RF) supervised analysis of creatinine- and body weight–standardized metabolite patterns from 2015 by sex groups (M=male, F=female). (A–B) show results of PLS-DA, with score plot in (A) and permutation testing in (B). (C) shows results for RF performance.

5. Supplemental Table S2. Demographic factors of the study subset reporting current employment who were asked about bolis consumption.

6. Data. Raw metabolic and risk factor data provided. Metadata included (.xlsx).
