# Supplementary material for: Urinary Metabolomic Profile of Youth at Risk of Chronic Kidney Disease in Nicaragua
Source: Kidney360. 2023 Apr 17;4(7):899–908. doi: 10.34067/KID.0000000000000129 (PMC10371259; doi:10.34067/KID.0000000000000129)
Supplement: Supplementary file 1 [file kidney360-4-0899-s001.pdf]

SUPPLEMENTARY MATERIAL

Table of Contents

1. **Supplemental Methods.** Serum creatinine measurement for use in CKiD eGFR calculation.
2. **Supplemental Figure S1.** Flowchart of cohort participants.
3. **Supplemental Table S1.** Urine metabolites (n=50) measured for each participant through ^{1}H-NMR.
4. **Supplemental Figure S2.** Partial least square-discriminant analysis (PLS-DA) and random forest (RF) supervised analysis of creatinine- and body-weight-standardized metabolite patterns from 2015 by sex groups (M=male, F=female). Panels (a)-(b) show results of PLS-DA, with score plot in (a) and permutation testing in (b). Panel (c) shows results for RF performance.
5. **Supplemental Table S2.** Demographic factors of study subset reporting current employment who were asked about bolis consumption.
6. **Data:** Raw metabolic and risk factor data provided. Metadata included. (.xlsx)

Supplemental Methods. Serum creatinine measurement for use in CKiD eGFR calculation.

Serum creatinine (SCr) was measured at the National Center for Diagnosis and References Laboratory (CNDR), the Nicaraguan Ministry of Health's central diagnostic laboratory in Managua, using a COBAS 400, ROCHE automated analyzer.

Supplemental Figure S1. Flowchart of cohort participants.

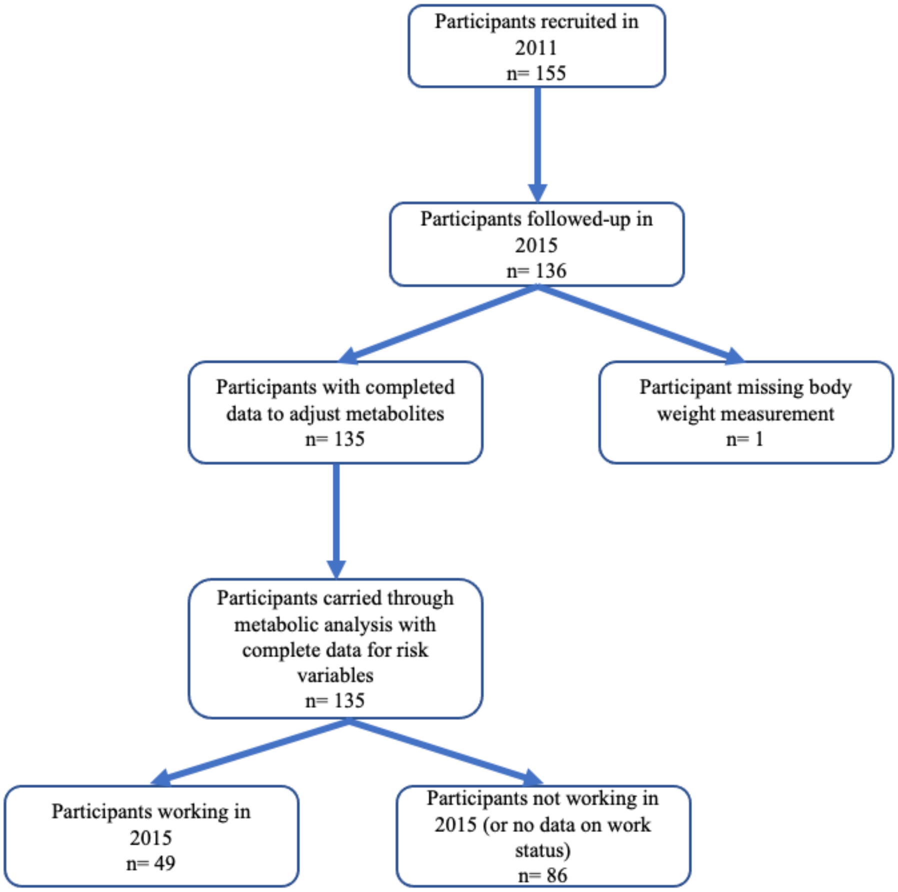

Supplemental Table S1. Urine metabolites (n=50) measured for each participant through ^{1}H-NMR.

Urine metabolites	
N6-methyladenosine	Mandelic acid
3-Methylhistidine	D-mannitol
1-Methylnicotinamide	D-mannose
Furoylglycine	Dimethylamine
Methylsuccinic acid	Formic acid
2-Oxoglutaric acid	Fumaric acid
Hydroxybutyric acid	Glycine
Gamma aminobutyric acid	Guanidinoacetic acid
Acetic acid	Hippuric acid
Acetoacetic acid	Imidazole
Acetone	Inosine
Adenosine	Lactic acid
Alanine	Methionine
Allantoin	Myoinositol
Allopurinol	Dimethylglycine
Arginine	Oxaloacetic acid
Benzoic acid	Proline betaine
Betaine	Pyruvic acid
Caffeine	Sarcosine
Citric acid	Succinic acid
Creatine	Tartaric acid
Creatinine	Taurine
D-galactose	Trigonelline
D-glucose	Trimethylamine
Lactose	Valine

Supplemental Figure S2. Partial least square-discriminant analysis (PLS-DA) and random forest (RF) supervised analysis of creatinine- and body-weight-standardized metabolite patterns from 2015 by sex groups (M=male, F=female). Panels (a)-(b) show results of PLS-DA, with score plot in (a) and permutation testing in (b). Panel (c) shows results for RF performance.

(a)

(b)

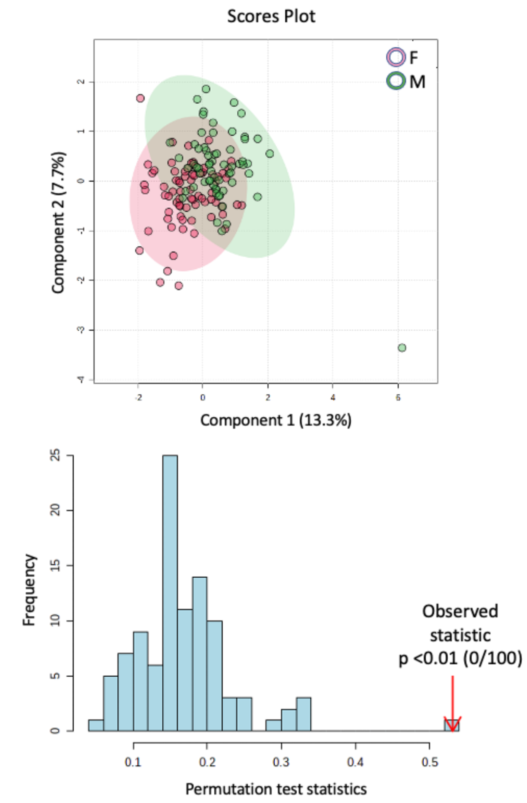

(c)

	F	M	Class Error	Out-of-bag Error	Matthew's Correlation Coefficient
F	61	12	0.16	0.213	0.57
M	17	46	0.27		

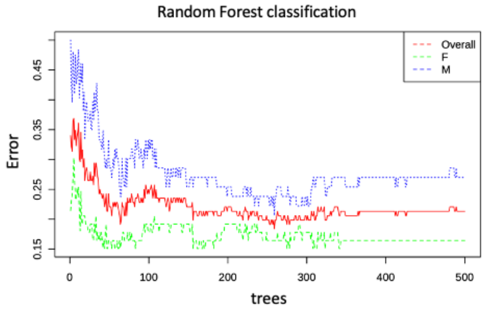

Supplemental Table S2. Demographic factors of study subset reporting current employment who were asked about bolis consumption.

	Study Participants Working in 2015 (n=50)
Sex	
Male (n, %)	28/50 (56%)
Body weight, kg (median, IQR)	68.0 (25)
Age	
Range (in years)	14-22
Median, IQR (in years)	20 (2)
$\geq$18 years old (n,%)	45/50 (90%)
High-risk region for adult MeN[a] (n, %)	22/50 (44%)
Familial history of MeN[b]	5/50 (10%)
eGFR $\leq$90 mL/min/1.73 m^2	3/49 (6.1%)
Have consumed bolis at work[c]	11/49 (22.4%)
